# Management and Follow-Up of a Cohort of Neonates With Meconium Aspiration Syndrome Before and After the Revision of the 2015 Neonatal Resuscitation Program

**DOI:** 10.7759/cureus.72894

**Published:** 2024-11-02

**Authors:** Inês Silva Costa, Jose Alarcão, Ana Rodrigues Silva, Raquel Henriques

**Affiliations:** 1 Pediatrics Department, Unidade Local de Saúde (ULS) de Viseu Dão-Lafões, Viseu, PRT; 2 Neonatology Department, Unidade Local de Saúde (ULS) de Coimbra, Coimbra, PRT

**Keywords:** meconium, meconium aspiration syndrome (mas), neonatal intensive care unit (nicu), neonatology nrp neonatal resuscitation, respiratory support

## Abstract

Introduction: Meconium aspiration syndrome (MAS) is a respiratory condition associated with high morbimortality. Standards of care changed over the years addressing its specificities, aiming to decrease poor respiratory and neurologic long-term outcomes. This study aims to compare the practice and outcomes of MAS in a level III neonatal intensive care unit before and after the revision of the 2015 Neonatal Resuscitation Program (NRP).

Methods: A 15-year retrospective cohort of patients with MAS was assessed regarding perinatal management, clinical outcome, and neurodevelopmental follow-up.

Results: A total of 52 MAS occurred, 51.9% (n=27) male gender, median gestational age 40 weeks (IQR 39-40), and mean birth weight of 3395±503 g. Recommendations of the NRP were implemented with a significant change in the management in the delivery room, as positive pressure ventilation was more frequent (p=0.001). No significant change was found in the clinical span or morbidity of MAS after NRP, except for lower acidosis (pH 7.0 vs. 7.2; p=0.042) and lower hyperlactatemia (12.2 vs. 6.4 mmol/l; p=0.035). Overall acute complications included pulmonary hypertension (21.2%, n=11) and pneumothorax (15.4%, n=8). To date, morbidity during follow-up didn't differ after NRP concerning global development delay (p=0.591), neurologic sequelae (p=0.276), and recurrent bronchospasm (p=0.614), in contrast with speech delay, which was less frequent in the later subgroup (p=0.023).

Conclusions: SAM persists as a concerning condition. From our data, the NRP showed no inferiority in the clinical outcomes, consistent with the literature but with higher frequency. Late morbidity is still a problem concerning cerebral palsy and global development delay, similar to known data.

## Introduction

Meconium aspiration syndrome (MAS) is a clinical condition that occurs when a newborn (NB) inhales a mixture of meconium-stained amniotic fluid, leading to respiratory distress. Understanding MAS and its implications is crucial to providing appropriate management for affected infants [1].

The presence of meconium in the amniotic fluid can be a sign of fetal distress, occurring when hypoxia or acidosis is present [1]. Factors such as post-term pregnancy, fetal growth restriction, or oligoamnios can increase the risk of meconium being released into the amniotic fluid [2]. The severity of symptoms in MAS is variable. Mild cases may only exhibit increased work of breathing in the first hours of life, while severe cases can present with significant respiratory distress with the need for mechanical ventilation. Diagnosis is typically based on clinical signs, a history of meconium-stained amniotic fluid, and compatible findings on chest X-ray [1-2].

The treatment of MAS primarily focuses on managing respiratory distress and providing supportive care. The infant may require oxygen therapy to improve oxygenation and ventilation. Antibiotics may also be administered to prevent or treat any associated infections. Additionally, careful attention is given to the baby's temperature regulation, nutrition, and electrolyte imbalance alterations [1-2]. Despite advances in neonatal care, MAS can still lead to complications, mostly air-leaking conditions such as pneumothorax and persistent pulmonary hypertension of the NB. The majority of infants with MAS fully recover, but attention to neurodevelopment should be a concern as the incidence of cerebral palsy and global development delay is still non-neglectable in this group of patients [1-2].

Management in the delivery room has changed over the years. Before 2015, non-vigorous infants were immediately intubated, and tracheal aspiration was performed through direct laryngoscopy. After the revision of the 2015 Neonatal Resuscitation Program (NRP), non-vigorous NB with inadequate heart rate or tonus started receiving immediate positive pressure ventilation in order not to delay adequate ventilation potentially caused by the previously recommended airway procedures [3].

This study aims to characterize the clinical span and follow-up of a cohort of patients diagnosed with MAS, particularly regarding the comparison between outcomes after the revision of the NRP.

## Materials and methods

We performed a retrospective, longitudinal analysis of a cohort of patients diagnosed with MAS who were admitted to a level III neonatal intensive care unit (NICU) for 15 years, between 1st January 2008 and 31st December 2022. The Ethics Committee of Centro Hospitalar e Universitário de Coimbra approved the study (approval number: 2023).

Patients were eligible for inclusion in the study when four cumulative criteria were met: being born through meconium-stained amniotic fluid, presenting signs of respiratory distress in the first hours of life, having radiological findings compatible with MAS (patchy infiltrates, hyperinflation areas, or consolidation/atelectasis), and the absence of an alternative explanation for that clinical condition.

Resuscitation methods, clinical outcomes, and acute and long-term complications were assessed for the primary analysis. Afterward, we aimed to identify whether changes in clinical outcome variables were of note after the implementation of the NRP.

The cohort was submitted for follow-up analysis for neurodevelopmental alterations or recurrent sibilance. Eligibility for follow-up included a minimum of 24 months for neurologic sequelae, 24 months for global development delay, and four years for speech delay. Recurrent bronchospasm was assumed when patients were followed in general pediatrics appointments for that reason or when three or more emergency department admissions in one year had a final diagnosis of bronchospasm.

Quantitative variables were expressed as absolute and relative frequencies and continuous variables as central tendency and dispersion measures according to their distribution. For the test hypothesis analysis, the association between quantitative variables was assessed by the chi-squared test. Continuous variables underwent normality testing with the Shapiro-Wilk and Kolmogorov-Smirnov tests, and afterward, a t-test for independent samples or a Mann-Whitney U test was applied to the sample.

Statistical analysis was performed with SPSS Statistics version 26.0 (IBM Corp. Released 2019. IBM SPSS Statistics for Windows, Version 26.0. Armonk, NY: IBM Corp.), assuming statistically significant values when p<0.05.

## Results

During the 15-year study period, 52 MAS occurred per 38,499 live births, 32 (0.14%) before NRP, and 20 (0.12%) after NRP. The perinatal clinical characteristics are represented in Table 1. There were no post-term pregnancies recorded. Three NB were born in level II hospitals and admitted to our unit after transfer due to the need for intensive care.

**Table 1:** Clinical characteristics of mothers, NBs, and deliveries per subgroup of the NRP IQR: interquartile rate, SD: standard deviation, CTR: cardiotocography record, NBs: newborns, NRP: 2015 Neonatal Resuscitation Program * chi-squared test, ^&^ Mann-Whitney U test, ^#^ t-test for independent samples

Perinatal characteristics	Before NRP	After NRP	p-value
Gestational age (weeks) median (IQR)	39.5 (39.0; 40.0)	40.0 (39.0; 40.0)	0.843^&^
C-section delivery n (%)	16 (50.0%)	10 (50.0%)	1,000*
Male sex n (%)	17 (53.1%)	10 (50.0%)	0.826*
Birth weight (grams) mean (SD)	3411.6 ± 95.2	3368.8 ± 100.9	0.768^#^
Apgar 1^st^ minute median (IQR)	5.0 (3.0; 6.0)	4.0 (3.0;7.0)	0.872^&^
Apgar 5^th^ minute median (IQR)	8.0 (7.0; 9.0)	8.0 (6.0; 8.0)	0.554^&^
Apgar 10^th^ minute median (IQR)	9.0 (8.0; 10.0)	9.0 (8.0; 9.0)	0.467^&^
Mothers age (years) mean (SD)	31.7 ± 0.8	29.8 ± 1.4	0.218^#^
Acute fetal distress n (%)	14 (43.8%)	11 (55.0%)	0.430*
Fetal growth restriction n (%)	5 (15.6%)	0 (0.0%)	0.063*
Pre-eclampsia n (%)	2 (6.3%)	1 (5.0%)	0.851*
Oligoamnios n (%)	3 (9.4%)	1 (5.0%)	0.565*
Membrane rupture >18h n (%)	4 (12.5%)	3 (15.0%)	0.797*

Management in the delivery room was significantly different before and after the NRP (Table 2), as initial positive pressure ventilation was more frequent in the later subgroup (p<0.001; OR: 8.556, CI: 95% (2.177; 33.629)), and aspiration through direct laryngoscopy was not common practice as the first standard approach (p=0.025; OR: 0.556, CI: 95% (0.428; 0.721)). Regarding ventilatory support, no differences were seen in the need for mechanical ventilation, days of ventilation, or maximum FiO2 needed.

**Table 2:** Clinical management in the delivery group, chosen therapeutic conduct, and complications per subgroup of the NRP EET: endotracheal intubation, IQR: interquartile rate, SD: standard deviation, NRP: 2015 Neonatal Resuscitation Program * chi-squared test, ^&^ Mann-Whitney U test, ^# ^t-test for independent samples

Clinical management	Before NRP	After NRP	p-value
Management in the delivery room			
Visualized tracheal meconium n (%)	21 (65.6%)	10 (50.0%)	0.264*
Initial positive pressure ventilation n (%)	4 (12.5%)	11 (55.0%)	0.001*
Aspiration in EET and extubation n (%)	7 (21.9%)	0 (0.0%)	0.025*
Ressuscitation with EET n (%)	22 (68.8%)	13 (65.0%)	0.779*
Therapeutics			
Mechanical ventilation n (%)	21 (65.6%)	14 (70.0%)	0.744*
Days of mechanical ventilation median (IQR)	2.0 (1.0; 5.0)	5.0 (1.7; 6.3)	0.145^&^
Maximum required FiO_2_ (percentage) mean (SD)	63.0 ± 6.0	54.6 ± 5.8	0.941^#^
Nitric oxide n (%)	5 (15.6%)	3 (15.0%)	0.952*
Surfactant administration n (%)	13 (40.6%)	7 (35.0%)	0.685*
Inotropes n (%)	1 (3.1%)	3 (15.0%)	0.118*
Therapeutic hypothermia n (%)	4 (12.5%)	1 (5.0%)	0.372*
Immediate complications			
pH in venous blood 1 hour after delivery median (IQR)	7.0 (6.9; 7.2)	7.2 (7.1; 7.3)	0.042^&^
Lactacidemia (mmol/l) mean (SD)	12.2 ± 4.6	6.4 ± 4.2	0.035^#^
Pneumothorax n (%)	5 (15.6%)	3 (15.0%)	0.768*
Hipoxico-ischemic encephalopathy n (%)	7 (21.9%)	4 (20.0%)	0.872*
Seizures n (%)	4 (12.5%)	2 (10.0%)	0.784*
Pulmonary hypertension n (%)	4 (12.5%)	7 (35.0%)	0.053*
Early neonatal sepsis n (%)	2 (6.3%)	1 (5.0%)	0.851*

From the group of NBs with suspected asphyxia, six met the criteria for therapeutic hypothermia. One NB didn´t start hypothermia as his clinical condition worsened and he died. Data for pH in venous blood samples were available for 37 NBs and lactate serum levels for 27 NBs.

Regarding long-term follow-up (Table 3), five NBs from the subgroup before the NRP were lost to follow-up, and 27 patients (84.4%) were to be included for final analysis. The subgroup of NB after NRP consisted of one NB who died in the early neonatal period (1.9%), and follow-up protocol was applied for 14 NB for language development delay, 16 for recurrent bronchospasm, and 18 for global development delay and neurologic sequelae.

**Table 3:** Clinical outcomes during long-term follow-up per subgroup of the NRP NRP: 2015 Neonatal Resuscitation Program * chi-squared test

Follow-up outcomes	Before NRP	After NRP	p*
Global development delay n (%)	3 (11.1%)	3 (16.7%)	0.591
Neurologic sequelae n (%)	1 (3.7%)	2 (11.1%)	0.276
Speech delay n (%)	8 (29.6%)	0 (0.0%)	0.023
Recurrent bronchospasm n (%)	5 (18.5%)	4 (25.0%)	0.614

Speech delay was the only clinical condition significantly different after the NRP, with decreasing frequency (p=0.023; OR: 0.372, CI: 95% (0.252; 0.549)).

## Discussion

MAS remains an important pathology in the neonatal period responsible for many NICU admissions in our setting. A slight decrease in MAS frequency has occurred since 2015, which may be attributed to the improvement in perinatal practices, both obstetric and neonatal. However, it is important to note that both MAS incidences in this case series, before and after NRP (0.14% vs. 0.12%), are lower than the published data. This strongly suggests that the late efforts to prevent and manage MAS showed to be effective. There is still room for further research and improvement in reducing its occurrence and associated risks for NBs [1-4].

No post-term pregnancies were recorded, a recognized risk factor for MAS, which aligns with current obstetric practices. Cochrane reviews show that elective induction at 41 weeks of gestation reduces the risk of perinatal mortality with a lower incidence of MAS in the induced group (relative risk: 0.29, CI: 95% (0.12-0.68)). This risk factor continues to be more prevalent in developing countries [5]. The prevalence of other risk factors such as non-reassuring cardiotocography records, oligoamnios, and fetal growth restriction is similar to several studies, although cohorts in our country report a lower frequency of these potential hazards [1-2,4-6]. These clinical findings should raise particular concern about the approach to the NB in the delivery room, as they often correlate with the risk for hypoxia or acidosis, both strongly associated with MAS. If prenatal risks are present, a trained team of resuscitators should be available to receive the NB [7].

The significant change in practice performance in the delivery room is in line with the adoption of the new recommendations of the NRP, with good acceptance of the new guidelines among professionals in the service. The need for resuscitation with EET was similar in both NRP subgroups, before and after 2015. This data supports that although preventive actions are required, the need for resuscitation with EET remained, in contrast with the change of the initial airway approach [2,6-8]. The reported death rate among MAS ranges from 1.2% to 7% and is higher in developing countries [1-2,4]. Our rate is overlapping and concordant with higher mortality rates in NBs with pulmonary hypertension and asphyxia, as they were both complications in the clinical condition of the NB who died in our sample [1].

Regarding the therapeutic adjuvants, about 50% of NBs will require exogenous surfactant or inhaled nitric oxide [8]. In our study group, we observed a lower utilization of these practices, and there is no robust evidence to recommend them routinely in every patient with MAS [9].

MAS is known to be linked with significant morbidity and mortality. In terms of pulmonary complications, approximately 15-33% of patients with MAS experience air leaks, such as pneumothorax or pneumomediastinum, and 4% present seizures [1-2,7]. Our cohort has heterogeneous results, with a lower frequency of pneumothorax but a higher frequency of seizures. Hypoxic-ischemic encephalopathy was less frequent than the 30% described in many cohorts [4,10-11].

Acidosis and hyperlacticaemia are associated with MAS, and their decrease after NRP is in concordance with less NB meeting the criteria for therapeutic hypothermia. This improvement may be attributed to reduced exposure to potential hazards prior to delivery.

Long-term outcomes, namely the neurological prognosis, are notably poor, as approximately 21% of infants experience cerebral palsy or global developmental delay [1-2]. This indicates that predisposing and underlying factors contributing to MAS are fundamental in the development of neurodevelopmental impairments among the surviving infants, regardless of the mode of delivery, the severity of the illness, or the ventilation method utilized [12-13]. Our cohort presented neurological outcomes that match the inferior limit of frequency in most studies. The decrease in the incidence of speech delay may result from a more holistic approach to NB care, focusing on long-term neurodevelopment rather than solely immediate outcomes. However, it may also reflect a lower threshold for introducing supportive and developmental measures during the growth of infants, as they are more influenced by their environment than by interventions primarily aimed at motor impairments like cerebral palsy, which are less preventable. Follow-up for speech delay has also a bias since our cohort after NRP was only achieved for infants born prior to 2019. On respiratory outcomes, wheezing could be expected to affect up to 50% of infants but was less than half of the predicted values [2,10-11].

Our study has limitations, including the retrospective characteristics of the data and the number of NB included in the cohort. Attention should also be given to the case definition, which excludes patients with mild respiratory distress who are solely receiving supplementary oxygen and may have been excluded due to the absence of radiological criteria. This case definition may potentially lead to a higher inclusion rate of NBs with severe MAS.

## Conclusions

Overall, our study concludes that there has been no worsening of immediate or long-term outcomes after the implementation of NRP. This emphasizes the idea that the previous recommendations, with more invasive maneuvers in non-vigorous NB, do not substantially impact the immediate clinical condition of the patient. For this reason, less invasive care in this subgroup of NB should continue to be the standard practice until further evidence arises. Studies with longer follow-up periods and focusing on preventive strategies are needed to support new recommendations.
